# Migration distance as a selective episode for wing morphology in a migratory insect

**DOI:** 10.1186/s40462-017-0098-9

**Published:** 2017-04-05

**Authors:** D. T. Tyler Flockhart, Blair Fitz-gerald, Lincoln P. Brower, Rachael Derbyshire, Sonia Altizer, Keith A. Hobson, Leonard I. Wassenaar, D. Ryan Norris

**Affiliations:** 1 Department of Integrative Biology, University of Guelph, Guelph, ON N1G 2W1 Canada; 2 Department of Biology, Sweet Briar College, Sweet Briar, VA 24595 USA; 3 Odum School of Ecology, University of Georgia, Athens, GA 30602 USA; 4 Environment Canada, Saskatoon, SK S7N 3H5 Canada; 5 Department of Biology, University of Western Ontario, London, ON N6A 5B7 Canada; 6 International Atomic Energy Agency, Department of Nuclear Sciences and Applications, Vienna, A-1400 Austria

**Keywords:** Seasonal migration, Stable isotopes, Deuterium, Monarch butterfly, *Danaus plexippus*, Survival

## Abstract

**Background:**

Selective pressures that occur during long-distance migration can influence morphological traits across a range of taxa. In flying insects, selection should favour individuals that have wing morphologies that increase energy efficiency and survival. In monarch butterflies, differences in wing morphology between migratory and resident populations suggest that migratory populations have undergone selection for larger (as measured by length and area) and more elongated (as measured by roundness and aspect ratio) forewings. However, selection on wing morphology may also occur within migratory populations, particularly if individuals or populations consistently migrate different distances.

**Results:**

Using 613 monarch butterflies that were collected on the Mexican wintering grounds between 1976 – 2014, we tested whether monarch wing traits were associated with migratory distance from their natal areas in eastern North America (migration range: 774–4430 km), as inferred by stable-hydrogen (*δ* ^2^H) and -carbon (*δ* ^13^C) isotopic measurements. Monarchs that migrated farther distances to reach their overwintering sites tended to have longer and larger wings, suggesting positive selective pressure during migration on wing length and area. There was no relationship between migration distances and either roundness or aspect ratio.

**Conclusions:**

Our results provide correlative evidence that the migratory period may act as a selective episode on monarch butterfly wing morphology, although selection during other portions of the annual cycle, as well as extensive mixing of individuals from various natal locations on the breeding grounds, likely counteracts directional selection of migration on morphology.

**Electronic supplementary material:**

The online version of this article (doi:10.1186/s40462-017-0098-9) contains supplementary material, which is available to authorized users.

## Background

The selective pressures associated with long-distance migration can influence morphology in a variety of taxa, including birds, insects and fish [1–4]. Because migration is energetically expensive [3] and high mortality is predicted during the migratory phase of the annual cycle [5, 6], selection should favour individuals that have morphological advantages that increase efficiency and hence migratory success. For example, populations of birds that migrate longer distances have longer and more pointed wings than sedentary populations or populations that migrate shorter distances [7–11]. Long and thin wings with elongated tips improve aerodynamics and reduce drag during gliding flight [12, 13]. The same selection pressures should operate on migratory insects [4, 14], but examining this hypothesis is hampered by the challenges associated with assigning insect origins and tracking long-distance movements.

One way to estimate migratory distance of insects is through the use of endogenous markers such as stable isotopes [15, 16]. Stable isotope relative abundances (e.g. *δ* ^2^H, *δ* ^15^N and *δ* ^13^C) are translated up the food chain [15, 17] and, if they vary in a spatially predicable manner (termed *isoscapes*; [18]), reflect the geographic origin of where tissues are grown in herbivores and higher order consumers [15]. Since stable isotopes in the chitin of wing tissue of adult flying insects is metabolically inert after formation [15, 19, 20], stable isotope measurements can be used to identify the geographic natal origin of adults regardless of how far they have flown from the natal site [21]. Stable isotope measurements have been used successfully to determine natal origin of migratory insects such as butterflies [22–24] and dragonflies [21].

Monarch butterflies (*Danaus plexippus*) in eastern North America are known for their fall migration up to 5000 km from their northernmost breeding range in Canada to overwintering sites in central Mexico [25]. Migratory monarchs are known to have longer wings compared to resident populations [26–28]. Individuals with more elongated wings tended to be seen earlier in migration than those with short wings, suggesting that elongated wings increase the speed of migration [29]. At overwintering grounds in Mexico [30] and the west coast of the U.S. [31], there is a negative correlation between hydrogen isotope values (*δ* ^2^H) and wing size, suggesting that individuals with larger wings derived from more northern locations and so migrate longer distances. These studies were based on single-year sampling, and recent work showed that patterns of monarch natal origin can vary annually [32]. Furthermore, both studies used *δ* ^2^H values as a proxy for migration distance, which, in some cases, can introduce a significant source of variation because *δ* ^2^H values do not necessarily correlate directly with distance travelled. At the same time, larger adult wing sizes may arise when larvae develop at lower mean temperatures at northern latitudes [33] and this parameter must be controlled for in statistical analyses.

**Fig. 1:**
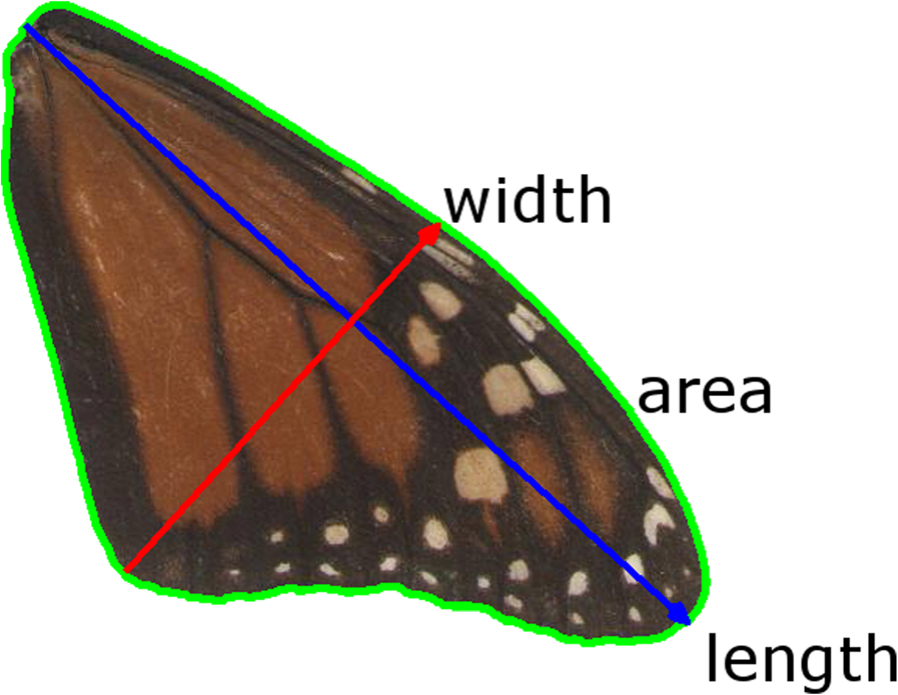
The forewing measurements made for each butterfly in ImageJ [38] include the length (*l*), width (*w*), and surface area (*a*). From these measurements we calculated the roundness 4(*a*/(π*l*)^2^) and aspect ratio (*l*/*w*). Measurements followed that of [26] and [37] (for roundness)

Here we examined the hypothesis that migration distance acts as a selective episode on monarch wing morphology because of the energetic costs of longer flights. To examine this hypothesis, we estimated migration distance of overwintering monarchs in Mexico sampled over five decades by estimating natal origin from two stable isotopes (*δ* ^13^C and *δ* ^2^H) in their wing tissue [22, 34–36]. Given that last generation monarchs in eastern North America migrate directly to a few spatially clustered locations in central Mexico [37], fall migration distance and ambient temperature experienced during larval development can be estimated as a direct function of natal origin in eastern North America. We considered that individuals sampled in Mexico to be representative of those that survived the migratory journey. Thus, if migration distance influenced wing morphology by selecting for individuals with more efficient long-distance flight [26], after controlling for temperature we predicted that fall migration distance would be positively related to *i*) forewing size (as measured by length and area; Fig. 1) and *ii*) the degree of elongation in the forewing (as measured by roundness and aspect ratio; Fig. 1) in monarchs sampled on their overwintering grounds.

## Methods

### Butterfly collections and wing morphology measurement

Monarch butterflies (*Danaus plexippus*) (*N* = 613) from the eastern North American migratory population were collected from the Sierra Chincua Sanctuary in Mexico during the overwintering period (November - February) from 1976–2015; samples from 1988 did not have any colony location recorded (Additional file 1: Table S1). Forewings were detached from body using forceps and scanned on an HP PSC 750 flatbed scanner at 300 dots per inch [38]. ImageJ software [39] was used to take wing morphology measurements (length, width, area) of forewings images following Altizer and Davis [26]. Length was defined as the point of attachment at the thorax to the distal tip of the wing, width was defined as the longest line drawn perpendicular to the length axis, and area was defined as the total surface area of the forewing (Fig. 1). These measurements were then used to calculate wing roundness (4 × [area/(π × length)^2^]; [38]) and aspect ratio (length/width; [26]). Our measurements of roundness reduced the skew of perimeter measurements that occurred from variance due to wing wear [38] and resulted in data that were normally distributed in order to meet the assumptions of linear models. Individuals with wings that were frayed or damaged that would have resulted in inaccurate forewing length measurements were excluded from our analyses (*N* = 91). The distance measurements using the centroid method (see below) did not vary between the butterflies included and those excluded (*t* = −1.19, *p* = 0.24). However, in a pattern opposite to our expectations, butterflies with worn wings (mean distance: 2900 km) had shorter migration distances using the high point method (see below) compared to butterflies with pristine wings (3104 km; *t* = −2.13, *p* = 0.04).

### Stable H and C isotope analysis

Butterflies were stored in glassine or paper envelopes and frozen or stored in desiccators until analysis. Wing tissue membrane was washed twice in 2:1 chloroform:methanol solution to remove surface oils and contaminants. Wing chitin subsamples (1.0 ± 0.1 mg) for *δ* ^13^C were loaded into 8.0 mm × 5.0 mm pressed tin capsules and combusted online using a Eurovector 3000 (Milan, Italy – www.eurovector.it) elemental analyzer. The resulting CO_2_ and N_2_ gases were separated by gas chromatography (GC) and introduced into a Nu Horizon (Nu Instruments, Wrexham, UK – www.nu-ins.com) triple-collector isotope-ratio mass-spectrometer via an open split and compared to a pure CO_2_ or N_2_ reference gas. Wing chitin subsamples (0.35 ± 0.02 mg) for *δ* ^2^H isotopes were loaded into 4.0 mm × 3.2 mm silver capsules, loaded into a zero-blank carousel and combusted using flash pyrolysis (1350 °C). Hydrogen gas was separated in a Eurovector Elemental Analyzer (Milan, Italy) and isotopic measurements made using an interfaced Elementar Isoprime™ (Isoprime, Manchester, UK) Continuous Flow Isotope Mass Spectrometer (CFIRMS). Non-exchangeable hydrogen *δ* ^2^H values for monarch wing chitin were obtained using the Comparative Equilibrium procedure [40], with results normalized to the VSMOW-SLAP scales. Laboratory keratin standards were equilibrated with the lab atmosphere for 72 h along with samples. The assigned values for hydrogen isotopes were CBS and KHS standards, currently having *δ* ^2^H_VSMOW_ values of −197‰ and −54‰, respectively. Within-run reproducibility (*n* = 5) for *δ* ^2^H values of keratin standards were better than ± 2‰. Laboratory standards for *δ* ^13^C were BWBII and PUGEL with assigned *δ* ^13^C_VPDB_ values of −18.5‰ and −13.6‰ vs. the VPDB primary standard. The within-run precision of laboratory keratin control standards (n = 5) were better than ± 0.15‰ for *δ* ^13^C. All analyses were conducted at the Stable Isotope Hydrology and Ecology Laboratory, Environment Canada, Saskatoon, Saskatchewan.

### Migratory distance measurements

Migratory distance was defined as the distance between collection location at the overwintering colonies in Mexico and the isotopically assigned natal origin of each monarch using *δ* ^13^C and *δ* ^2^H measurements. Details of probabilistic assignment of natal origin are found in [32] but we briefly summarize the approach below. We used multivariate normal distribution assignment models to calculate the probability of natal origin to each pixel in a continuous landscape that spans the monarch breeding distribution [34, 36, 41]. For each butterfly, the model calculated the probability of natal origin to each pixel in our study area based on the correspondence between *δ* ^2^H and *δ* ^13^C values in wing tissue to the isoscape-predicted values of monarch *δ* ^2^H and *δ* ^13^C wing tissue of each geographically indexed cell in the landscape [42].

The probability density of individual *i* having location *j* as the natal origin is *Y*_*i*_ ~ *N*(*μ*_*j*_, *Σ*) where *Y*_*i*_ is a vector of observed *δ* ^2^H and *δ* ^13^C values, *μ*_*j*_ is a vector of predicted *δ* ^2^H and *δ* ^13^C values derived from calibrated isoscapes [32], and Σ is the positive-definite variance-covariance matrix of *δ* ^2^H and *δ* ^13^C. Here, Σ was assumed to be constant across the entire isoscape and was estimated based on all values from known-location butterflies from data in [15]. We applied Bayes’ rule to invert the conditional probabilities of natal origin based on isotopes as:

$$ {f}_{J\Big| Y, X, M}\ \left( J= j\Big| Y = {y}_{ij}, X = {x}_j\right) = \frac{f_{Y\Big| X}\ \left( Y = {y}_{ij}\Big| X = {x}_j\right)\ {f}_{J\Big| M}\ \left( J= j\Big| M\right)}{{\displaystyle {\sum}_j}{f}_{Y\Big| X}\left( Y = {y}_{ij}\Big| X = {x}_j\right){f}_{J\Big| M}\left( J= j\Big| M\right)} $$

where f_J|Y,X,M_ is the spatially explicit posterior probability density function for location *j* as the true origin of individual with measured isotope value *y*, given the measured isotope values *y*_*ij*_ for locations *x*_*j*_. The function f_Y|X_ represents the conditional distribution on *Y*_*j*_ from above. The function f_J|M_ is the probability of occurrence for locations *J*, using a distributional prior of the breeding distribution, M, as described in [32].

From this probabilistic surface we calculated that latitude and longitude of natal origin in two ways. The high point method considered the latitude and longitude of the pixel with the highest probability to be the natal origin; we present the statistical results using the high point method in the main text of the manuscript. The centroid method calculated the mean latitude and mean longitude of the natal origin based on the odds that a given assigned origin was correct relative to the odds that it was incorrect as 2:1 and coded the upper 33% of the assignment surface for each butterfly as a binary surface [43]. The odds ratio constitutes the compromise between having sufficient geographic structure in the assignments while correctly assigning the natal origin of an individual [44], and is akin to choosing a type I error rate (e.g. α = 0.05) in a traditional statistical test to determine significance. The centroid method accounts for uncertainty in the assignment of natal origin and reduces the potential effects of natal origins that are assigned near the edge of the breeding distribution based on a limited geographic extent of the isoscapes. The statistical results using the centroid method are presented in the supplementary material. Migration distance was calculated as a straight-line distance from the latitude and longitude of the assigned natal origins to the Sierra Chincua colony (19°40’20.6”N, 100°17’42.6”W; [45]).

### Weather data

Temperature-dependent development in monarchs may result in butterflies from northern areas developing at slower rates than southern areas [32]. If so, then controlling for temperature at the natal location in statistical analysis would allow us to account for environmental effects on wing morphology. Daily maximum temperature data for North America was downloaded [46, 47] and we calculated the mean maximum daily temperature between July 20 and August 9, which is the development period for overwintering butterflies when they are larvae (following Flockhart et al. [32]). For each butterfly, we extracted the mean maximum daily temperature value at both the high point and centroid location. To assess the effects of collinearity, we calculated the variance inflation factor for each variable in generalized linear models [48]. Variance inflation factors assess the boosted standard errors of parameters in generalized linear models which may obscure significant relationships between explanatory and response variables. The variance inflation factors of the variables in our models were generally lower than 3 (Additional file 1: Table S2) which is the recommended value when analysts should to take action to address collinearity [48] so we so ran analyses (see below) both with and without mean maximum daily temperature.

### Statistical analysis

To examine how migration distance influenced wing morphology, we constructed global linear mixed effects models using the lme4 package [49] in program R for each response variable: forewing area, forewing length, roundness, and aspect ratio. These global models included sex and migration distance as fixed effects and year as a random effect. Sex was included because males had larger wings than females [26, 27]. We included year as a random effect because selection pressures likely change each year based on regional temperature-dependent development schedules that influence breeding distribution [36, 50–52] and autumn weather patterns that influence survival during migration, but these conditions were not expected to change in a predictable pattern over time. In a repeated analysis we also included mean maximum daily temperature as a fixed factor.

After constructing each global model, we removed the distance parameter for each response variable, and compared the reduced model to the global model using a likelihood ratio test [53]. This allowed us to evaluate whether the distance parameter significantly improved the model fit. We also ran the same series of global models as generalized linear models, substituting the Southern Oscillation Index (SOI) for year as a fixed effect in the model. SOI is a large-scale measure of climate that would influence all monarchs similarly in a given year and has a significant influence on the assigned natal origins of monarchs overwintering in Mexico [32]. Model outputs from the generalized linear models, as well as all models that include mean maximum daily temperature at the natal location, raw data, and R code are found in the Additional files 1, 2, and 3.

## Results

Monarchs in our study were estimated to have migrated between 774 km and 4430 km from natal locations to overwintering sites. Migratory distance was correlated with *δ* ^2^H values but this relationship was not linear (Additional file 1: Figure S1), suggesting that our two-isotope assignment approach was likely a better estimate of migration distance than using *δ* ^2^H values alone. Mean maximum daily temperature at natal origins ranged between 15.1–38.3 °C and, as expected, migratory distance and mean maximum daily temperature were correlated (high point: *r* = −0.78; centroid: *r* = −0.8; Additional file 1: Figure S1).

**Fig. 2:**
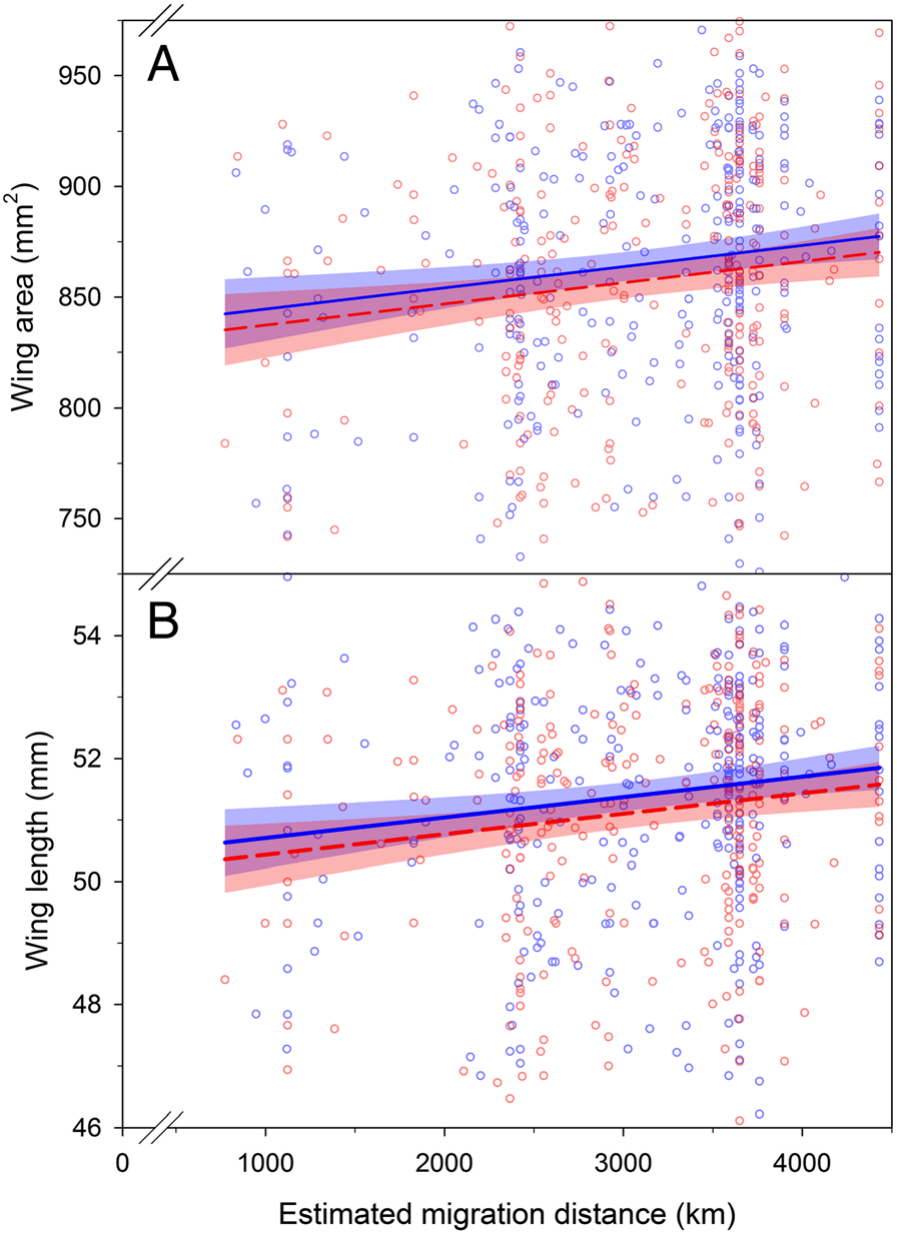
Wing area (**a**) and wing length (**b**) of monarch butterflies that successfully migrated to overwintering areas plotted against the migratory distance between natal origins and the Sierra Chincua overwinter colony in Mexico. The mean (line) and 95% confidence interval (shading) are the model-estimated relationship for females (red dashed line) and males (blue solid line). Raw data are presented as points for females (red) and males (blue). Note the reduced range of the y-axis is shown to depict the relationships for wing area (range: 636 – 1027 mm^2^) and wing length (range: 42.7–57.3 mm)

There were no significant differences between males and females in migration distance (*t* = −0.01, *p* = 0.99). On average, male monarchs had longer forewings and greater forewing area than females, but males and females had similar wing roundness and aspect ratios (Additional file 1: Table S2). Using mixed-effects models that included year as a random effect (Additional file 1: Figure S2), both male and female monarchs that migrated longer distances had wings that were longer and had larger wing area (Table 1, Fig. 2). Although the parameter estimates were in the predicted direction, monarchs that migrated longer distances did not have more pointed wings or larger aspect ratios (Table 1). The direction of the parameter estimates and statistical inference between migratory distance and wing morphology was consistent when considering these relationships with generalized linear models that quantified annual variation using global oscillations indices (Additional file 1: Table S3). Global oscillation indices were not significant predictors of wing area (*t* = 0.57, *p* = 0.57), wing length (*t* = 1.47, *p* = 0.14) or aspect ratio (*t* = 1.73, *p* = 0.08) but were negatively related to wing roundness (β = −0.0008, *t* = 3.17, *p* = 0.001). Results using the high point method were consistent with the centroid method for estimating migration distance in both mixed-effects (Additional file 1: Table S4) and generalized linear models (Additional file 1: Table S5).

**Table 1:** Results of likelihood ratio tests used to test the effect of migration distance on wing morphology using the high point method

Response variable	χ^2^	df	p	Distance parameter estimate	95% confidence interval
Wing Area	5.62	1	0.02	0.0124	0.0022, 0.0226
Wing Length	5.20	1	0.02	3.966e-4	5.577e-5, 7.374e-4
Roundness	1.99	1	0.159	3.084e-6	−1.21e-6, 7.38e-6
Aspect Ratio	0.19	1	0.659	1.89e-6	−6.52e-6, 1.03e-5

For each wing morphology response variable, a global linear mixed effects model was constructed with sex, mean maximum daily temperature and migration distance as fixed effects, and year as a random effect. Distance was then removed from each model and compared to the global model using a likelihood ratio test. The *χ* ^2^ test statistic was used to calculate the p-value for each likelihood ratio test

Variance inflation factors were generally less than 3 indicating that collinearity would have limited effects on drawing inference on the effects of migration distance on wing morphology (Additional file 1: Table S2). After controlling for mean maximum daily temperature, monarchs that migrated longer distances had wings that were longer and had larger wing area but there was no effect of migration distance on wing roundness and aspect ratio (Additional file 1: Tables S6). Using generalized linear models, there was no effect of mean maximum daily temperature on any wing attribute but the effect of migration distance on wing area and length remained significant (Additional file 1: Tables S7). There was no effect of migration distance on wing roundness or aspect ratio (Additional file 1: Tables S7). Similar to above, after controlling for mean maximum daily temperature the results using the high point method were consistent with the centroid method for estimating migration distance in both mixed-effects (Additional file 1: Table S8) and generalized linear models (Additional file 1: Table S9).

## Discussion

Consistent with previous studies [26, 28, 30, 31], our results showed that monarchs that migrated the farthest distance on average from natal grounds to wintering sites had the longest and largest wings. This observation suggests that migration acted as a selective episode on monarch butterfly wing morphology, specifically on wing length and area, and this relationship did not arise from temperature-dependent effects on wing morphology. Our study extended previous findings by proposing a mechanism by which natural selection may operate on wing morphology in migratory populations – longer and larger wings lead to higher survival of monarch butterflies during long-distance migration. In other words, monarchs that initiate fall migration from the most extreme limit of their range are more likely to successfully reach their wintering sites if they have longer wings and greater wing surface area, whereas shorter winged monarchs with small wing surface area have a greater chance of successful migration if they start from locations closer to the wintering sites. This result appeared to be consistent over the 40-year time series of monarchs collected in Mexico.

Efficient flight is important for small insects flying thousands of kilometers between breeding and non-breeding areas, and for which lipid accumulation during long distance movement is important for migration and overwinter survival [3, 54]. During migration, monarchs use both powered and gliding flight [55, 56]. Powered flight is more efficient with long wings and gliding/soaring flight is more efficient with reduced wing loading (ratio of mass:wing area; [13]). While we did not measure wing loading directly, we found a positive relationship between migration distance and forewing area, one component of wing loading. Moreover, prior work showed that wing area is strongly positively correlated to both body length (from head to abdomen) and total body mass of monarchs [26], suggesting that monarchs with larger wing area are also larger in body size overall. Thus, work here provides partial evidence that selection might operate on monarch wing length and area that influences wing loading during migration. This finding has also been reported for studies on migratory birds, which found a relationship between migration distance, wing morphology and body size at both the genus [7, 10] and species [8, 9, 11] levels. Skeletal morphology that promotes efficient travel has also been reported for salmon that migrated long distances [2], illustrating that selection for efficient travel during migration is important in non-flying migrants as well.

There was no evidence that wing roundness and aspect ratio correlated with migratory distance in our sample of monarchs, although the parameter estimates measured here were in the predicted direction of greater distance being associated with more angular (less round) wings. In a cross-population comparison, Davis and Altizer [26] found that non-migratory monarchs from Central America, Florida and the Caribbean have rounder (less elongated) forewings than migratory monarchs from eastern North America. Moreover, Dockx [27] showed that among monarchs captured in Cuba, migrants originating from northern latitudes had more angular wings than those identified as year-round residents. In more recent work, however, Li et al. [28] found no difference in wing shape among monarchs from migratory versus non-migratory populations, and Altizer et al. [30] also found no relationship between wing shape and estimated migration distance among eastern North American migrants. Thus, evidence for selection operating on wing shape in migratory monarchs appears to be mixed.

While evidence suggests that wing traits linked to migration in monarchs are genetically heritable [26], it is important to note that the fitness benefit of longer and larger wings could vary over different portions of the monarch’s annual cycle or even after arriving at the overwintering colonies. Monarchs overwinter in dense colonies of millions of individuals with specific microclimatic conditions [57]. Clusters of butterflies retain heat, and monarchs in the middle of clusters are more sheltered from extreme temperatures and moisture on their wings that can freeze and kill them [57, 58]. Theoretically, larger monarchs might compete better for positions near the core of clusters; this advantage could be bolstered by earlier arrival times at the overwintering colonies. On the other hand, high conspecific density in colonies [up to 50 million butterflies per hectare [57] may result in large individuals having a disadvantage for obtaining an optimal roosting location, which requires precise and controlled flight in confined spaces. Smaller males are also more likely to mate at the overwintering sites than larger males [59], although this appears to be caused more by small males initiating mating attempts as opposed to a selective advantage conferred by small wings, as captive experiments showed that males with larger wings mated more often than small males [60]. Additionally, monarchs breed over successive generations [36, 51] and summer migratory distances must be significantly shorter than the fall migration distances and hence selection may not be as strong during the breeding season. Wing measurements of butterflies collected during the breeding season coupled with migratory distance estimates derived from stable isotopes would inform whether selection operates during the breeding season.

Individuals with larger wings might experience lower wing loading and greater flight efficiency during long-distance migration [12, 61]. On the other hand, Borland et al. [62] found no difference in the wing length of butterflies captured during migration through the USA in Minnesota versus Texas, which suggests that if smaller individuals were dying during migration, this happened after monarchs entered Mexico [54]. If so, then we would also predict that monarchs that remigrate from Mexico to Texas to breed would also have longer wings than those migrating through Texas the previous autumn. Given the prediction that longer wings should increase survival of monarchs during migration, we would also predict that larger butterflies could amass great fat reserves to fuel the long overwintering period. Monarchs in particular accumulate fat reserves during that last portion of migration between Texas and overwintering colonies in Mexico [63]. From this perspective, there could be a complex interaction among wing loading, lipid reserves and successful migration.

Although results here indicate that long-distance migration selects for larger wings, we are not suggesting that this will necessarily result in the evolution of longer-winged sub-populations within the eastern migratory population. Since monarchs of all migratory distances overwinter together each winter and breed in the spring, any effect of wing size selection will be limited by population mixing during migration itself and at the overwintering sites [22, 31, 64–66]. Moreover, because of the multiple-generation aspect of monarch migration, individuals do not usually return and breed in their natal region [35], making it unlikely that the same selection pressures will act on the genetic line of longer-distance migrants in the following migratory season. Therefore, although the migratory population in eastern North America is morphologically different from non-migratory ones in other parts of the world, it is unlikely that eastern monarchs will experience directional selection on wing morphology. The recurring selective disappearance of individuals with shorter wings during autumn migration arises from a dilution of the selection pressures acting on wing morphology across multiple breeding generations. If this dilution effect operates in other multivoltine insects, then migratory behaviour may be less likely to evolve in insects compared to vertebrate taxa.

## Conclusions

Understanding the determinants of successful migration during the annual cycle could improve understanding of how populations will respond to future conditions. For example, over the coming century, the distribution of both monarchs and their host plants are predicted to move northward due to climate change [67, 68]. If this bears out, these distributional changes will require individuals to successfully complete longer autumn migrations to traditional overwintering areas in Mexico. The prospect that individuals with longer wings have an advantage during long-distance migration suggests that at least some monarchs should be able to conduct this longer migration to overwintering locations in Mexico in the future. Although monarchs may colonize and successfully breed in more northern areas in coming decades, biologists should not assume that that will automatically enhance or stabilize population persistence if these same morphologies are more strongly selected against at different portions of the annual cycle. Future work might well consider how competing selective forces of flight maneuverability, thermoregulation, and migration distance interact to influence wing morphology and the consequences for population distribution of the monarch butterfly.

## Additional files

Additional file 1: Figures S1 and S2, Table S1–S9. Additional results. (DOCX 6879 kb)

Additional file 2: The data set used in the analysis. (XLSX 801 bytes)

Additional file 3: The R code to run the analysis. (DOCX 14 kb)

## References

[1] Miller-Butterworth CM, Jacob DS, Harley EH (2003). Strong population substructure is correlated with morphology and ecology in a migratory bat. Nature.

[2] Crossin GT, Hinch SG, Farrell AP, Higgs DA, Lotto AG, Oakes JD, Healey MC (2004). Energetics and morphology of sockeye salmon: effects of upriver migratory distance and elevation. J Fish Biol.

[3] Bowlin MS, Wikelski M (2008). Pointed wings, low wingloading and calm air reduce migratory flight costs in songbirds. PLoS ONE.

[4] Johansson F, Söderquist M, Bokma F (2009). Insect wing shape evolution: independent effects on migratory and mate guarding flight in dragonfly wings. Biol J Linn Soc.

[5] Muir WD, Smith SG, Williams JG, Hockersmith EE, Skalski JR (2001). Survival estimates for migrant yearling Chinook salmon and steelhead tagged with passive integrated transponders in the Lowe Snake and Lower Columbia Rivers, 1993–1998. N Am J Fish Managem.

[6] Sillett TS, Holmes RT (2002). Variation in survivorship of a migratory songbird throughout its annual cycle. J Anim Ecol.

[7] Voelker G (2001). Morphological correlates of migratory distance and flight display in the avian genus *Anthus*. Biol J Linn Soc.

[8] Arizaga J, Campos F, Alonso D (2006). Variations in wing morphology among subspecies might reflect different migration distances in Bluethroat. Ornis Fennica.

[9] Baldwin MW, Winkler H, Organ CL, Helm B (2010). Wing pointedness associated with migratory distance in common-garden and comparative studies of stonechats (*Saxicola torquata*). J Evol Biol.

[10] Nowakowski JK, Szulc J, Remisiewicz M (2014). The further the flight, the longer the wing: relationship between wing length and migratory distance in Old World reed and bush Warblers (*Acrocephalidae* and *Locustellidae*). Ornis Fennica.

[11] Corman A, Bairlein F, Schmaljohann H (2014). The nature of the migration route shapes physiological traits and aerodynamic properties in a migratory songbird. Behav Ecol Sociobiol.

[12] Dudley R (1991). Biomechanics of flight in neotropical butterflies: Aerodynamics and mechanical power requirements. J Experim Biol.

[13] Wooton RJ (1992). Functional morphology of insect wings. Ann Rev Entomol.

[14] Roff DA, Fairbairn DJ (2007). The evolution and genetics of migration in insects. Bioscience.

[15] Hobson KA, Wassenaar LI, Taylor OR (1999). Stable isotopes (δD and δ^13^C) are geographic indicators of natal origins of monarch butterflies in eastern North America. Oecologia.

[16] Rubenstein DR, Hobson KA (2004). From birds to butterflies: animal movement patterns and stable isotopes. Trend Ecol Evol.

[17] Flockhart DTT, Kyser TK, Chipley D, Miller NG, Norris DR (2015). Experimental evidence shows no fractionation of strontium isotopes (^87^Sr/^86^Sr) among soil, plants, and herbivores: implications for tracking wildlife and forensic science. Isotopes Environ Health Studies.

[18] West JB, Bowen GJ, Dawson TE, Tu KP (2010). Isoscapes.

[19] Brattström O, Wassenaar LI, Hobson KA, Åkesson S (2008). Placing butterflies on the map – testing regional geographical resolution of three stable isotopes in Sweden using the monophagus peacock *Inachis io*. Ecography.

[20] Hobson KA, Soto DX, Paulson DR, Wassenaar LI, Matthews JH (2012). A dragonfly (*δ*^2^H) isoscape for North America: a new tool for determining natal origins of migratory aquatic emergent insects. Meth Ecol Evol.

[21] Hobson KA, Anderson RC, Soto DX, Wassenaar LI (2012). Isotopic evidence that dragonflies (*Pantala flavenscens*) migrating through the Maldives come from the northern Indian subcontinent. PLoS ONE.

[22] Wassenaar LI, Hobson KA (1998). Natal origins of migratory monarch butterflies at wintering colonies in Mexico: new isotopic evidence. Proc Natl Acad Sci U S A.

[23] Brattström O, Wassenaar LI, Hobson KA, Åkesson S (2010). Understanding the migration ecology of European red admirals *Vanessa atalanta* using stable hydrogen isotopes. Ecography.

[24] Stefansecu C, Soto DX, Talavera G, Vila R, Hobson KA (2016). Long-distance autumn migration across the Sahara by painted lady butterflies: exploiting resource pulses in the tropical savannah. Biol Lett.

[25] Urquhart FA, Urquhart NR (1978). Autumnal migration routes of the eastern population of the monarch butterfly (*Danaus p. plexippus* L; Danaidae; Lepidoptera) in North America to the overwintering site in the Neovolcanic Plateau of Mexico. Can J Zool.

[26] Altizer S, Davis AK (2010). Populations of monarch butterflies with different migratory behaviors show divergence in wing morphology. Evolution.

[27] Dockx C (2007). Directional and stabilizing selection on wing size and shape in migrant and resident monarch butterflies, *Danaus plexippus* (L.), in Cuba. Biol J Linn Soc.

[28] Li Y, Pierce AA, de Roode JC (2016). Variation in forewing size linked to migratory status in monarch butterflies. Animal Migration.

[29] Satterfield D, Davis AK (2014). Variation in wing characteristics of monarch butterflies during migration: Earlier migrants have redder and more elongated wings. Animal Migration.

[30] Altizer S, Hobson KA, Davis AK, de Roode JC, Wassenaar LI (2015). Do healthy monarchs migrate farther: Tracking natal origins of parasitized vs. uninfected monarch butterflies overwintering in Mexico. PLoS ONE.

[31] Yang LH, Ostrovsky D, Rogers MC, Welker JM (2016). Intra-population variation in the natal origins and wing morphology of overwintering western monarch butterflies *Danaus plexippus*. Ecography.

[32] Flockhart DTT, Brower LP, Ramirez MI, Hobson KA, Wassenaar LI, Altizer S, Norris DR. Regional climate on breeding grounds predicts variation in the natal origin of monarch butterflies overwintering in Mexico over 38 years. Global Change Biology, 2017; doi:10.1111/gcb.13589. 10.1111/gcb.13589 28045226

[33] Kingsolver JG, Huey RB (2008). Size, temperature, and fitness: three rules. Evol Ecol Res.

[34] Miller NG, Wassenaar LI, Hobson KA, Norris DR (2011). Monarch butterflies cross the Appalachians from the west to recolonize the east coast of North America. Biol Lett.

[35] Miller NG, Wassenaar LI, Hobson KA, Norris DR (2012). Migratory connectivity of the monarch butterfly (*Danaus plexippus*): Patterns of spring re-colonization in Eastern North America. PLoS ONE.

[36] Flockhart DTT, Wassenaar LI, Martin TG, Hobson KA, Wunder MB, Norris DR (2013). Tracking multi-generational colonization of the breeding grounds by monarch butterflies in eastern North America. Proc Roy Soc Lond B Biol Sci.

[37] Vidal O, Rendón-Salinas E (2014). Dynamics and trends of overwintering colonies of the monarch butterfly in Mexico. Biol Conserv.

[38] Hanley D, Miller NG, Flockhart DTT, Norris DR (2013). Forewing pigmentation predicts migration distance in wild-caught migratory monarch butterflies. Behav Ecol.

[39] Rasband WS. ImageJ, *U. S. National Institutes of Health*, Bethesda, Maryland, USA, http://imagej.nih.gov/ij/. 1997.

[40] Wassenaar LI, Hobson KA (2003). Comparative equilibration and online technique for determination of non-exchangeable hydrogen of keratins for use in animal migration studies. Isot Environ Health Studies.

[41] Royle JA, Rubenstein DR (2004). The role of species abundance in determining breeding origins of migratory birds with stable isotopes. Ecol Appl.

[42] Wunder MB. Using isoscapes to model probability surfaces for determining geographic origins. In: West JB, Bowen GJ, Dawson TE, Tu KP, editors. Isoscapes: understanding movement, patterns and process on earth through isotope mapping. Netherlands: Springer; 2010. p. 251–270.

[43] Hobson KA, Wunder MB, Van Wilgenburg SL, Clark RG, Wassenaar LI (2009). A method for investigating population declines of migratory birds using stable isotopes: origins of harvested lesser scaup in North America. PLoS ONE.

[44] Hobson KA, Van Wilgenburg SL, Wassenaar LI, Larson K (2012). Linking hydrogen (*δ*^2^H) isotopes in feathers and precipitation: sources of variance and consequences for assignment to isoscapes. PLoS ONE.

[45] Brower LP, Slayback DA, Jaramillo-López P, Ramirez IM, Oberhauser KS, Williams EH, Fink LS (2016). Illegal logging of 10 hectares of forest in the Sierra Chincua monarch butterfly overwintering area in Mexico. Am Entomol.

[46] Maurer EP, Wood AW, Adam JC, Lettenbaier DP, Nijssen B (2002). A long-term hydrologically-based data set of land surface fluxes and states for the conterminous United States. J Climate.

[47] Thornton PE, Thornton MM, Mayer BW, Wei Y, Devarakonda R, Wilhelmi N. 2016. Daymet: daily surface weather data on a 1-km grid for North America, Version 3. Data set available on-line [http://daac.ornl.gov] from Oak Ridge National Laboratory Distributed Active Archive Center, Oak Ridge, Tennessee, USA. Date accessed: 2017/02/09. Temporal range: 1980/01/01-2015/12/31. http://dx.doi.org/10.3334/ORNLDAAC/1328.

[48] Zuur AF, Ieno EN, Elphick CS (2010). A protocol for data exploration to avoid common statistical problems. Meth Ecol Evol.

[49] Bates D, Maechler M, Bolker B, Walker S (2015). Fitting linear mixed-effects models using lme4. J Stat Software.

[50] Malcolm SB, Cockrell BJ, Brower LP (1987). Monarch butterfly voltinism: effects of temperature constraints at different latitudes. Oikos.

[51] Cockrell BJ, Malcolm SB, Brower LP, Malcolm SB, Zalucki MP (1993). Time, temperature, and latitudinal constraints on the annual recolonization of eastern North America by the monarch butterfly. Biology and Conservation of the Monarch Butterfly.

[52] Feddema JJ, Shields J, Taylor OR, Bennett D, Oberhauser KS, Solensky MJ (2004). Simulating the development and migration of the monarch butterfly. Monarch Butterfly Biology & Conservation.

[53] Vuong QH (1989). Likelihood ratio tests for model selection and non-nested hypotheses. Econometrica.

[54] Brower LP, Fink LS, Kiphart RJ, Pocius V, Zubieta RR, Ramirez MI, Oberhauser KS, Nail KR, Altizer S (2015). Effect of the 2010–2011 drought on the lipid content of monarchs migrating through Texas to overwintering sites in Mexico. Monarchs in a Changing World: Biology and Conservation of an Iconic Butterfly.

[55] Gibo DL. Flight strategies of migrating monarch butterflies (*Danaus plexippus* L.) in southern Ontario. In: Danthanarayana W, editor. Insect Flight: Dispersal and Migration. Berlin Heidelberg: Springer; 1986. p. 172–184.

[56] Gibo DL, Pallett MJ (1979). Soaring flight of monarch butterflies *Danaus plexippus* (Lepidotpera: Danaidae), during the late summer migration in southern Ontario. Can J Zool.

[57] Brower LP, Williams EH, Fink LS, Zubieta RR, Ramirez MI (2008). Monarch butterfly clusters provide microclimatic advantages during the overwintering season in Mexico. J Lepidop Soc.

[58] Anderson JB, Brower LP (1996). Freeze-protection of overwintering monarch butterflies in Mexico: critical role of the forest as a blanket and an umbrella. Ecol Entomol.

[59] Van Hook T, Malcolm SB, Zalucki MP (1993). Non-random mating in monarch butterflies overwintering in Mexico. Biology and Conservation of the Monarch Butterfly.

[60] Davis AK, Cope N, Smith A, Solensky MJ (2007). Wing color predicts future mating success in male monarch butterflies. Ann Entomol Soc Am.

[61] Bradley CA, Altizer S (2005). Parasites hinder monarch butterfly flight: implications for disease spread in migratory hosts. Ecol Lett.

[62] Borland J, Johnson CC, Crumpton TW, Thomas M, Altizer SM, Oberhauser KS, Oberhauser KS, Solensky MJ (2004). Characteristics of fall migratory monarch butterflies, *Danaus plexippus*, in Minnesota and Texas. Monarch Butterfly Biology & Conservation.

[63] Brower LP, Fink LS, Walford P (2006). Fueling the fall migration of the monarch butterfly. Integrat Compar Biol.

[64] Eanes WF, Koehn RK (1978). An analysis of genetic structure in the monarch butterfly, *Danaus plexippus* L. Evolution.

[65] Lyons JI, Pierce AA, Barribeau SM, Sternberg ED, Mongue AJ, de Roode JC (2012). Lack of genetic differentiation between monarch butterflies with divergent migration destinations. Mol Ecol.

[66] Pfeiler E, Nazario-Yepiz NO, Pérez-Gálvez F, Chávez-Mora CA, Loustalot Laclette MR, Rendón-Salinas E, Markow TA. Population genetics of overwintering monarch butterflies, *Danaus plexippus* (Linnaeus), from central Mexico inferred from mitochondrial DNA and microsatellite markers. J Heredity 2016; doi:10.1093/jhered/esw071 10.1093/jhered/esw071 PMC5434545 28003372

[67] Batalden RV, Oberhauser K, Peterson AT (2007). Ecological niches in sequential generations of eastern North American monarch butterflies (Lepidoptera: Danaidae): The ecology of migration and likely climate change implications. Environ Entomol.

[68] Lemoine NP (2015). Climate change may alter breeding ground distributions of eastern migratory monarchs (*Danaus plexippus*) via range expansion of *Asclepias* host plants. PLoS ONE.

