# Supplementary material for: Migration distance as a selective episode for wing morphology in a migratory insect
Source: Mov Ecol. 2017 Apr 5;5:7. doi: 10.1186/s40462-017-0098-9 (PMC5381079; doi:10.1186/s40462-017-0098-9)
Supplement: Supplementary file 1 — Additional results. (DOCX 6879 kb) [file 40462_2017_98_MOESM1_ESM.docx]

**Additional File 1.**

**Migration distance as a selective episode for wing morphology in a migratory insect**

D. T. Tyler Flockhart, Blair Fitz-gerald, Lincoln P. Brower, Rachael Derbyshire, Sonia Altizer, Keith A. Hobson, Leonard I. Wassenaar, & D. Ryan Norris

Corresponding author: [dflockha@uoguelph.ca](mailto:dflockha@uoguelph.ca)

**
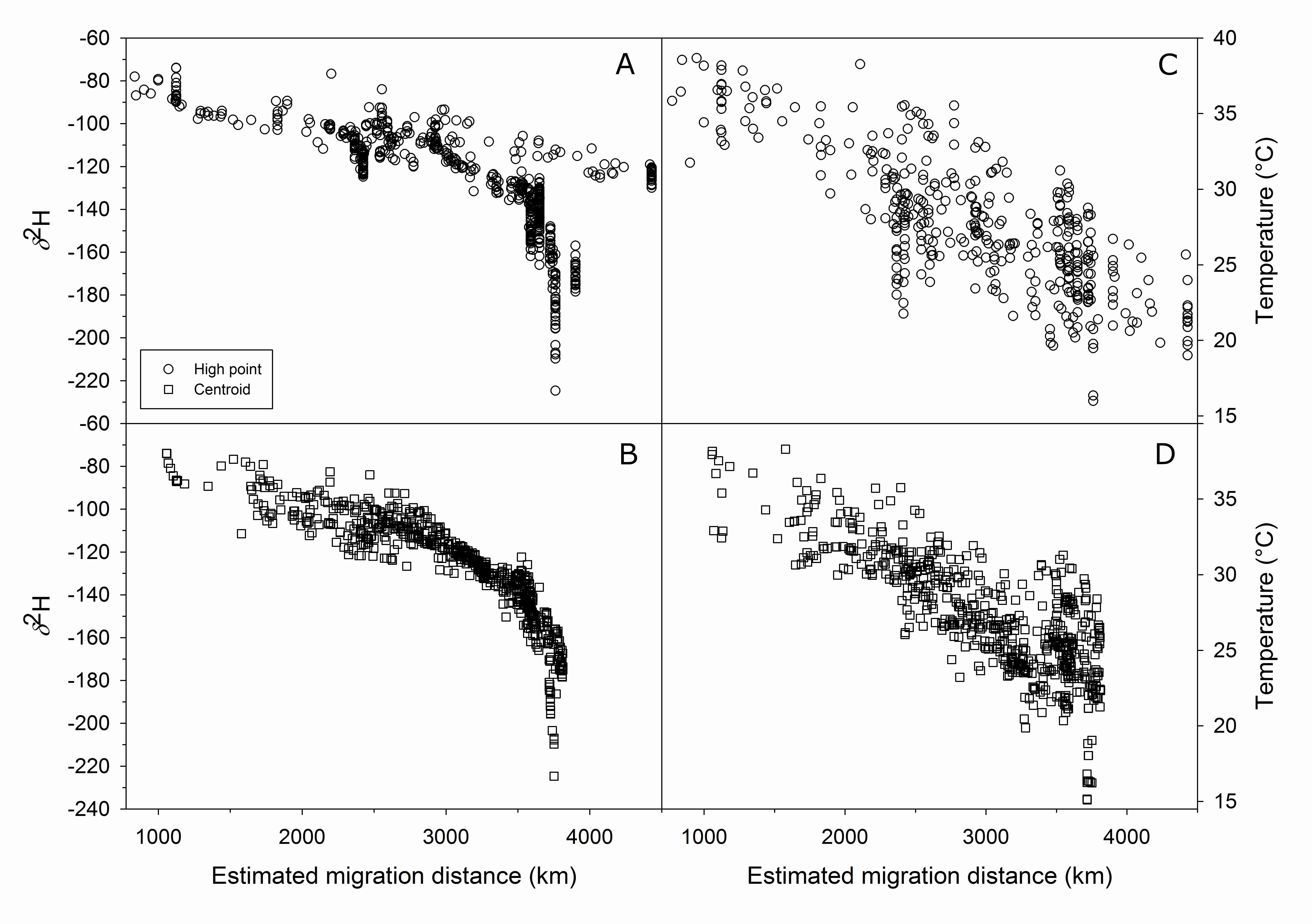
**

**Figure S1.** Correlations of the estimated migration distance (km) of monarchs and their stable-hydrogen isotope value (*δ*^2^H) using (A) the pixel with the highest probability of natal origin (*r* = -0.72, *p* < 0.001) and (B) the mean latitude and longitude of the centroid natal distribution (*r* = -0.86, *p* < 0.001). Correlation of the estimated migration distance (km) and mean temperature between July 20 and August 9 using (C) the pixel with the highest probability of natal origin (*r* = -0.78) and (D) the mean latitude and longitude of the centroid natal distribution (*r* = -0.80).


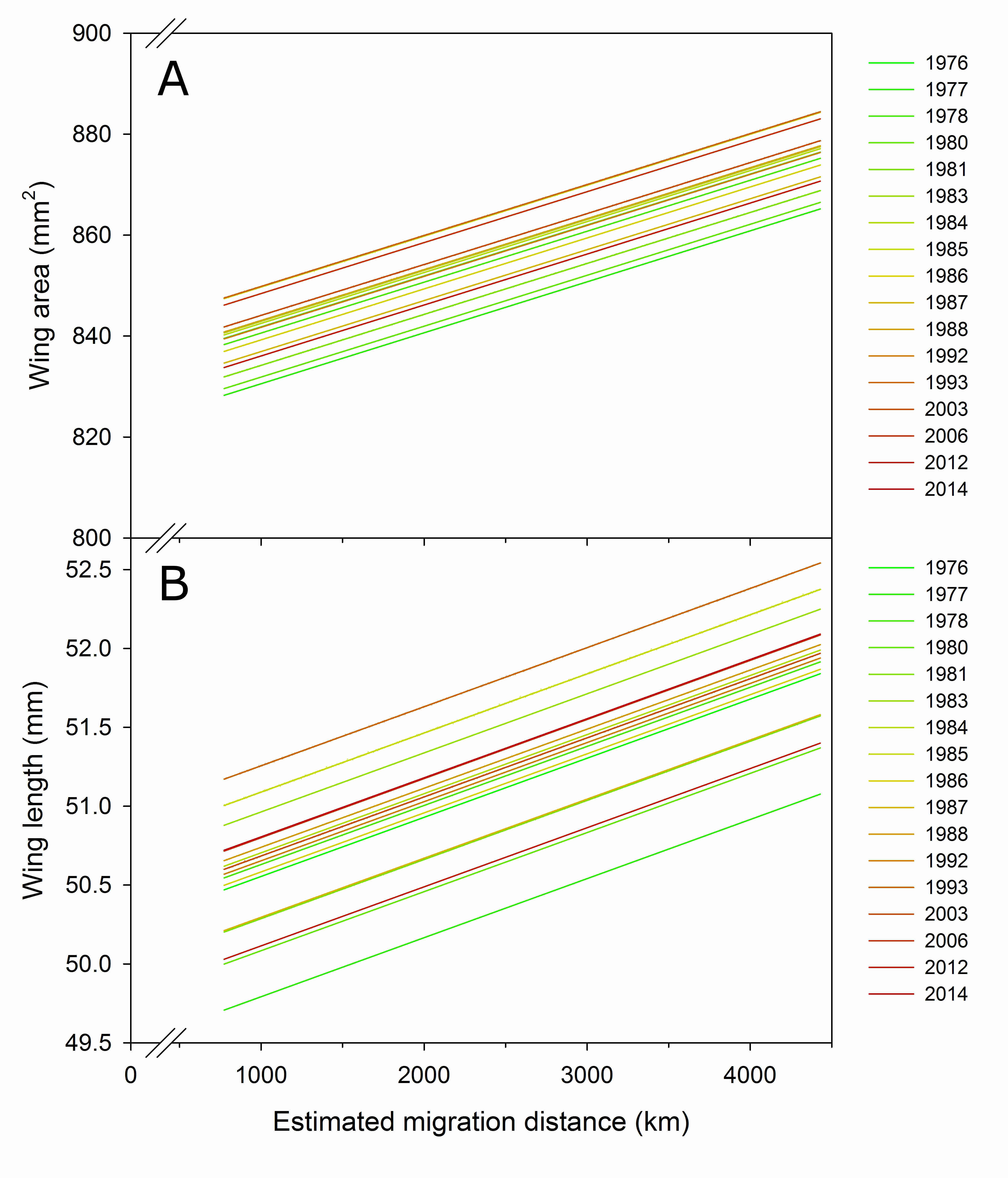


**Figure S2.** Random effects of year on the relationship between wing area (A) and wing length (B) length of monarch butterflies that successfully migrated to overwintering areas plotted against the migratory distance between breeding origins and the Sierra Chincua overwinter colony in Mexico.

**Table S1.** Mean and standard deviation of wing morphology measurements of male and female monarch butterflies collected over 17 years at the Sierra Chincua overwintering colony in central Mexico. The overwintering period is from November to March, so “year” is referenced to November. An “NA” for standard deviation occurs where n = 1.

|  | Wing length (mm) | | | |  | Wing area (mm) | | | |  | Roundness | | | |  | Aspect ratio | | | |  | Sample size | |
| --- | --- | --- | --- | --- | --- | --- | --- | --- | --- | --- | --- | --- | --- | --- | --- | --- | --- | --- | --- | --- | --- | --- |
|  | Female | | Male | |  | Female | | Male | |  | Female | | Male | |  | Female | | Male | |  | Female | Male |
| Year | mean | sd | mean | sd |  | mean | sd | mean | sd |  | mean | sd | mean | sd |  | mean | sd | mean | sd |  | N | N |
| 1976 | 49.41 | NA | 52.47 | NA |  | 823.1 | NA | 918.9 | NA |  | 0.137 | NA | 0.135 | NA |  | 1.88 | NA | 1.92 | NA |  | 1 | 1 |
| 1977 | 50.41 | 2.146 | 50.54 | 2.385 |  | 844.9 | 59.09 | 851.9 | 73.28 |  | 0.135 | 0.0043 | 0.135 | 0.0047 |  | 1.93 | 0.049 | 1.92 | 0.059 |  | 66 | 63 |
| 1978 | 51.27 | 2.017 | 51.30 | 1.034 |  | 855.4 | 65.32 | 853.7 | 33.53 |  | 0.132 | 0.0036 | 0.132 | 0.0021 |  | 1.95 | 0.048 | 1.96 | 0.040 |  | 7 | 8 |
| 1980 | 50.26 | 1.600 | 51.13 | 2.181 |  | 831.5 | 58.91 | 853.5 | 60.02 |  | 0.133 | 0.0038 | 0.132 | 0.0041 |  | 1.94 | 0.058 | 1.95 | 0.054 |  | 15 | 14 |
| 1981 | 51.01 | 1.969 | 51.09 | 2.001 |  | 848.7 | 64.61 | 851.7 | 55.66 |  | 0.132 | 0.0025 | 0.132 | 0.0034 |  | 1.96 | 0.027 | 1.96 | 0.037 |  | 19 | 18 |
| 1983 | 52.61 | 1.548 | 51.56 | 1.855 |  | 900.1 | 46.23 | 869.2 | 61.98 |  | 0.132 | 0.0032 | 0.132 | 0.0036 |  | 1.94 | 0.034 | 1.95 | 0.046 |  | 20 | 20 |
| 1984 | 50.83 | 1.997 | 52.00 | 2.011 |  | 849.1 | 59.23 | 874.1 | 58.97 |  | 0.133 | 0.0047 | 0.131 | 0.0050 |  | 1.94 | 0.059 | 1.95 | 0.052 |  | 19 | 20 |
| 1985 | 51.97 | 1.437 | 52.34 | 1.969 |  | 882.1 | 48.93 | 881.4 | 61.25 |  | 0.132 | 0.0026 | 0.130 | 0.0039 |  | 1.95 | 0.029 | 1.96 | 0.036 |  | 18 | 18 |
| 1986 | 51.69 | 2.252 | 50.85 | 1.909 |  | 871.3 | 61.00 | 839.7 | 61.40 |  | 0.132 | 0.0054 | 0.132 | 0.0046 |  | 1.94 | 0.049 | 1.95 | 0.055 |  | 17 | 19 |
| 1987 | 50.65 | 2.217 | 50.85 | 2.579 |  | 842.4 | 71.64 | 850.2 | 70.12 |  | 0.133 | 0.0034 | 0.133 | 0.0044 |  | 1.94 | 0.027 | 1.94 | 0.046 |  | 9 | 10 |
| 1988 | 51.51 | 1.046 | 51.53 | 2.373 |  | 848.7 | 47.98 | 873.6 | 62.94 |  | 0.130 | 0.0037 | 0.133 | 0.0049 |  | 1.98 | 0.045 | 1.93 | 0.050 |  | 12 | 20 |
| 1992 | 51.51 | 2.228 | 51.58 | 1.435 |  | 866.9 | 84.12 | 863.9 | 44.79 |  | 0.132 | 0.0041 | 0.132 | 0.0026 |  | 1.94 | 0.054 | 1.95 | 0.030 |  | 11 | 13 |
| 1993 | 52.00 | 1.866 | 52.52 | 2.048 |  | 874.8 | 58.04 | 881.8 | 66.03 |  | 0.131 | 0.0044 | 0.130 | 0.0050 |  | 1.96 | 0.051 | 1.98 | 0.060 |  | 17 | 19 |
| 2003 | 51.74 | 2.010 | 51.41 | 1.871 |  | 874.6 | 69.14 | 865.4 | 64.09 |  | 0.132 | 0.0027 | 0.133 | 0.0023 |  | 1.95 | 0.042 | 1.95 | 0.032 |  | 19 | 19 |
| 2006 | 51.08 | 1.697 | 52.15 | 2.330 |  | 856.1 | 54.47 | 895.8 | 68.92 |  | 0.133 | 0.0025 | 0.133 | 0.0049 |  | 1.94 | 0.034 | 1.94 | 0.062 |  | 17 | 19 |
| 2012 | 49.59 | 3.056 | 51.39 | 2.117 |  | 831.4 | 86.16 | 863.9 | 52.83 |  | 0.137 | 0.0081 | 0.133 | 0.0059 |  | 1.92 | 0.119 | 1.95 | 0.060 |  | 13 | 19 |
| 2014 | 51.56 | 1.796 | 51.85 | 2.050 |  | 854.5 | 55.98 | 876.3 | 62.38 |  | 0.130 | 0.0046 | 0.132 | 0.0030 |  | 1.96 | 0.055 | 1.94 | 0.053 |  | 15 | 18 |
| Total | 51.13 | 2.076 | 51.41 | 2.150 |  | 857.5 | 62.17 | 864.8 | 63.47 |  | 0.133 | 0.0044 | 0.133 | 0.0045 |  | 1.94 | 0.052 | 1.95 | 0.052 |  | 295 | 318 |

**Table S2**. Variance inflation factor (VIFs) of variables in generalized linear mixed models and generalized linear model. Generalized linear mixed effects models included sex, temperature and distance as fixed effects and year as a random effect. Generalized linear models included sex, temperature, distance and SOI as fixed effects.

| **Model** | **Response variable** | **Sex** | **Temperature** | **Distance** | **SOI** |
| --- | --- | --- | --- | --- | --- |
| *Distance measured using high point method* | | | | | |
| Mixed model | Wing area | 1.00 | 2.65 | 2.65 | NA |
|  | Wing length | 1.00 | 2.69 | 2.68 | NA |
|  | Roundness | 1.00 | 2.70 | 2.70 | NA |
|  | Aspect ratio | 1.00 | 2.66 | 2.65 | NA |
| Linear model | Wing area | 1.00 | 2.63 | 2.59 | 1.02 |
|  | Wing length | 1.00 | 2.63 | 2.59 | 1.02 |
|  | Roundness | 1.00 | 2.63 | 2.59 | 1.02 |
|  | Aspect ratio | 1.00 | 2.63 | 2.59 | 1.02 |
| *Distance measured using centroid method* | | | | | |
| Mixed model | Wing area | 1.00 | 2.93 | 2.93 | NA |
|  | Wing length | 1.00 | 3.03 | 3.03 | NA |
|  | Roundness | 1.00 | 3.08 | 3.08 | NA |
|  | Aspect ratio | 1.00 | 2.94 | 2.93 | NA |
| Linear model | Wing area | 1.01 | 2.81 | 2.77 | 1.02 |
|  | Wing length | 1.01 | 2.81 | 2.77 | 1.02 |
|  | Roundness | 1.01 | 2.81 | 2.77 | 1.02 |
|  | Aspect ratio | 1.01 | 2.81 | 2.77 | 1.02 |

**Table S3**: Results of linear models used to test the effect of migration distance using the high point method on wing morphology. In these models, the Southern Oscillation Index (SOI) was used as a fixed effect in place of year (random effect) in the linear mixed effects models.

| ***Global Model*** | | | | ***Distance parameter*** | | |
| --- | --- | --- | --- | --- | --- | --- |
| **Model** | **F-value** | ***df*** | ***p*** | **Estimate** | **95% CI** | ***p*** |
| Area = Sex + Distance + SOI | 3.86 | 3,609 | 0.009 | 0.0097 | 0.0034, 0.0160 | 0.002 |
| Length = Sex + Distance + SOI | 4.481 | 3,609 | 0.018 | 0.00034 | 0.00013, 0.00055 | 0.001 |
| Roundness = Sex + Distance + SOI | 2.237 | 3,609 | 0.083 | -2.39e-7 | -6.82e-7, 2.04e-7 | 0.290 |
| Aspect Ratio = Sex + Distance + SOI | 1.139 | 3,609 | 0.333 | 1.90e-6 | -3.27e-6, 7.06e-6 | 0.472 |

**Table S4**: Results of likelihood ratio tests used to test the effect of migration distance on wing morphology using the centroid method. For each wing morphology response variable, a linear mixed effects model was constructed with sex and distance as fixed effects, and year as a random effect. Distance was then removed from each model and compared to the global model using a likelihood ratio test. The χ^2^ test statistic was used to calculate the p-value for each likelihood ratio test.

| **Response variable** | ***χ*^2^** | ***df*** | ***p*** | ***Distance* parameter estimate** | **95% confidence interval** |
| --- | --- | --- | --- | --- | --- |
| Wing Area | 8.26 | 1 | 0.004 | 0.0117 | 0.0037, 0.0196 |
| Wing Length | 10.97 | 1 | <0.001 | 4.49e-4 | 1.84e-4, 7.14e-4 |
| Roundness | 1.84 | 1 | 0.175 | -3.84e-7 | -9.40e-7, 1.72e-7 |
| Aspect Ratio | 0.52 | 1 | 0.471 | 2.41e-6 | -4.14e-6, 8.97e-6 |

**Table S5**: Results of linear models used to test the effect of migration distance using the centroid method on wing morphology. In these models, the Southern Oscillation Index (SOI) was used as a fixed effect in place of year (random effect) in the linear mixed effects models.

| ***Global Model*** | | | | ***Distance* parameter** | | |
| --- | --- | --- | --- | --- | --- | --- |
| **Model** | **F-value** | ***df*** | ***p*** | **Estimate** | **95% CI** | ***p*** |
| Area = Sex + Distance + SOI | 3.187 | 3,609 | 0.0234 | 0.0108 | 0.00298,0.0187 | 0.007 |
| Length = Sex + Distance + SOI | 4.232 | 3,609 | 0.006 | 0.00039 | 0.000127,0.000653 | 0.004 |
| Roundness = Sex + Distance + SOI | 2.228 | 3,609 | 0.084 | -2.97e-7 | -8.52e-7,2.59e-7 | 0.30 |
| Aspect Ratio = Sex + Distance + SOI | 1.111 | 3,609 | 0.344 | 2.182e-6 | -4.31e-6,8.67e-6 | 0.51 |

**Table S6**: Results of likelihood ratio tests used to test the effect of migration distance on wing morphology using the high point method after accounting for the effect of temperature on wing morphology.

| **Response variable** | ***χ*^2^** | ***df*** | ***p*** | ***Distance* parameter** | | ***Temperature* parameter** | |
| --- | --- | --- | --- | --- | --- | --- | --- |
|  |  |  |  | **Estimate** | **95% confidence interval** | **Estimate** | **95% confidence interval** |
| Wing Area | 5.62 | 1 | 0.02 | 0.0124 | 0.0022, 0.0226 | 0.512 | -1.32, 2.33 |
| Wing Length | 5.20 | 1 | 0.02 | 3.966e-4 | 5.577e-5, 7.374e-4 | 0.0053 | -0.056, 0.066 |
| Roundness | 1.99 | 1 | 0.159 | 3.084e-6 | -1.21e-6, 7.38e-6 | 0.0003 | -0.0005, 0.0011 |
| Aspect Ratio | 0.19 | 1 | 0.659 | 1.89e-6 | -6.52e-6, 1.03e-5 | 6.632e-6 | -1.486e-3, 1.498e-3 |

For each wing morphology response variable, a linear mixed effects model was constructed with sex, temperature and distance as fixed effects, and year as a random effect. Distance was then removed from each model and compared to the global model using a likelihood ratio test. The χ^2^ test statistic was used to calculate the p-value for each likelihood ratio test.

**Table S7**: Results of linear models used to test the effect of migration distance using the high point method on wing morphology after accounting for the effect of temperature on wing morphology. In these models, the Southern Oscillation Index (SOI) was used as a fixed effect in place of year (random effect) in the linear mixed effects models.

| ***Global Model*** | | | | ***Distance* parameter** | | | ***Temperature* parameter** | | |
| --- | --- | --- | --- | --- | --- | --- | --- | --- | --- |
| **Model** | **F-value** | ***df*** | ***p*** | **Estimate** | **95% CI** | ***p*** | **Estimate** | **95% CI** | ***p*** |
| Area = Sex + Temperature + Distance + SOI | 3.211 | 4,606 | 0.013 | 0.0139 | 0.0039, 0.0239 | 0.007 | 0.93 | -0.83, 2.69 | 0.30 |
| Length = Sex + Temperature + Distance + SOI | 3.817 | 4,606 | 0.004 | 0.00047 | 0.00013, 0.00080 | 0.007 | 0.028 | -0.031, 0.087 | 0.35 |
| Roundness = Sex + Temperature + Distance + SOI | 2.846 | 4,606 | 0.023 | 8.96e-7 | -3.41e-6, 5.20e-6 | 0.683 | -4.27e-5 | -8.0e-4, 7.14e-4 | 0.91 |
| Aspect Ratio = Sex + Temperature + Distance + SOI | 0.809 | 4,606 | 0.520 | 2.195e-6 | -6.09e-6, 1.048e-5 | 0.603 | 9.69e-5 | -1.36e-3, 1.55e-3 | 0.90 |

**Table S8**: Results of likelihood ratio tests used to test the effect of migration distance on wing morphology using the centroid method. For each wing morphology response variable, a linear mixed effects model was constructed with sex, temperature and distance as fixed effects, and year as a random effect. Distance was then removed from each model and compared to the global model using a likelihood ratio test. The χ^2^ test statistic was used to calculate the p-value for each likelihood ratio test.

|  |  |  |  | ***Distance* parameter** | | ***Temperature* parameter** | |
| --- | --- | --- | --- | --- | --- | --- | --- |
| **Response variable** | ***χ*^2^** | ***df*** | ***p*** | **Estimate** | **95% confidence interval** | **Estimate** | **95% confidence interval** |
| Wing Area | 8.58 | 1 | 0.003 | 0.02031 | 0.0068, 0.03380 | 1.76 | -0.477, 3.977 |
| Wing Length | 8.60 | 1 | 0.003 | 6.87e-4 | 2.29e-4, 1.15e-3 | 0.048 | -0.028, 0.124 |
| Roundness | 0.54 | 1 | 0.464 | 2.18e-6 | -3.65e-6, 8.01e-6 | 0.00014 | -8.26e-4, 1.11e-3 |
| Aspect Ratio | 0.11 | 1 | 0.739 | 1.90e-6 | -9.31e-6, 1.31e-5 | -1.02e-4 | -1.94e-3, 1.73e-3 |

**Table S9**: Results of linear models used to test the effect of migration distance using the centroid method on wing morphology after accounting for the effect of temperature on wing morphology. In these models, the Southern Oscillation Index (SOI) was used as a fixed effect in place of year (random effect) in the linear mixed effects models.

| ***Global Model*** | | | | ***Distance parameter*** | | | ***Temperature* parameter** | | |
| --- | --- | --- | --- | --- | --- | --- | --- | --- | --- |
| **Model** | **F-value** | ***df*** | ***p*** | **Estimate** | **95% CI** | ***p*** | **Estimate** | **95% CI** | ***p*** |
| Area = Sex + Temperature + Distance + SOI | 3.443 | 4,608 | 0.0085 | 0.0216 | 0.0086,0.0346 | 0.001 | 2.187 | 0.082,4.291 | 0.042 |
| Length = Sex + Temperature + Distance + SOI | 4.143 | 4,608 | 0.003 | 0.00074 | 0.0003,0.0012 | 0.001 | 0.07 | -0.0004,0.1410 | 0.051 |
| Roundness = Sex + Temperature + Distance + SOI | 2.900 | 4,608 | 0.021 | -1.09e-6 | -6.69e-6, 4.50e-6 | 0.70 | -3.59e-4 | -1.26e-3,5.47e-4 | 0.44 |
| Aspect Ratio = Sex + Temperature + Distance + SOI | 0.832 | 4,608 | 0.505 | 2.326e-6 | -8.48e-6,1.31e-5 | 0.67 | 2.9e-5 | -1.72e-3,1.78e-3 | 0.97 |
