# Supplementary material for: Migration distance as a selective episode for wing morphology in a migratory insect
Source: Mov Ecol. 2017 Apr 5;5:7. doi: 10.1186/s40462-017-0098-9 (PMC5381079; doi:10.1186/s40462-017-0098-9)
Supplement: Supplementary file 3 — The R code to run the analysis. (DOCX 14 kb) [file 40462_2017_98_MOESM3_ESM.docx]

##################################################################

# Migration distance as a selective episode for wing morphology

# in a migratory insect

#

# Flockhart, Fitz-gerald, Brower, Derbyshire,

# Altizer, Hobson, Wassenaar, & Norris

#

# Submitted to Movement Ecology

#

# February 21, 2017

#

# The script analyzes the data using mixed-effects models and generalized

# linear models. Data are overwintered monarch butterflies collected from

# Mexico between 1976-2014. The migratory distance is the distance from the

# natal origin to the overwintering Chincua colony. Temperature is the mean

# maximum daily temperature (July 20 and August 9) at the natal origin

# location.

###################################################################

library(lme4)

library(car)

# Import data

MigDis<-read.csv("AdditionalFile2.csv")

# needed functions

vif.mer <- function (fit) {

## variance inflation factor; adapted from rms::vif

v <- vcov(fit)

nam <- names(fixef(fit))

## exclude intercepts

ns <- sum(1 * (nam == "Intercept" | nam == "(Intercept)"))

if (ns > 0) {

v <- v[-(1:ns), -(1:ns), drop = FALSE]

nam <- nam[-(1:ns)]

}

d <- diag(v)^0.5

v <- diag(solve(v/(d %o% d)))

names(v) <- nam

v

}

#############################################################################

################# Analyses without temperature

### Data Analysis using HIGH-POINT MIGRATION DISTANCE

## WingArea

# Mixed-effects models

waaH<-lmer(WingArea_mm~Sex+dist.high.point.km+(1|WinterYear), data=MigDis, REML=FALSE)

summary(waaH)

waaH2<-lmer(WingArea_mm~Sex+(1|WinterYear), data=MigDis, REML=FALSE)

summary(waaH2)

anova(waaH, waaH2, test="Chi")

confint(waaH)

# Analysis using linear models

wagmlH<-lm(WingArea_mm~Sex+dist.high.point.km+SOI, data=MigDis)

summary(wagmlH)

confint(wagmlH)

## WingLength

# Mixed-effects models

wlaH<-lmer(WingLength_mm~Sex+dist.high.point.km+(1|WinterYear), data=MigDis, REML=FALSE)

summary(wlaH)

wlaH2<-lmer(WingLength_mm~Sex+(1|WinterYear), data=MigDis, REML=FALSE)

summary(wlaH2)

anova(wlaH, wlaH2, test="Chi")

confint(wlaH)

# Analysis using linear models

wlgmlH<-lm(WingLength_mm~Sex+dist.high.point.km+SOI, data=MigDis)

summary(wlgmlH)

confint(wlgmlH)

## WingRoundness

# Mixed-effects models

r2aH<-lmer(Roundness~Sex+dist.high.point.km+(1|WinterYear), data=MigDis, REML=FALSE)

summary(r2aH)

r2aH2<-lmer(Roundness~Sex+(1|WinterYear), data=MigDis, REML=FALSE)

summary(r2aH2)

anova(r2aH, r2aH2, test="Chi")

confint(r2aH)

# Analysis using linear models

r2gmlH<-lm(Roundness~Sex+dist.high.point.km+SOI, data=MigDis)

summary(r2gmlH)

confint(r2gmlH)

## AspectRatio

# Mixed-effects models

araH<-lmer(AspectRatio~Sex+dist.high.point.km+(1|WinterYear), data=MigDis, REML=FALSE)

summary(araH)

araH2<-lmer(AspectRatio~Sex+(1|WinterYear), data=MigDis, REML=FALSE)

summary(araH2)

anova(araH, araH2, test="Chi")

confint(araH)

# Analysis using linear models

argmlH<-lm(AspectRatio~Sex+dist.high.point.km+SOI, data=MigDis)

summary(argmlH)

confint(argmlH)

### Data Analysis using CENTROID MIGRATION DISTANCE

## WingArea

# Mixed-effects models

waaC<-lmer(WingArea_mm~Sex+dist.centroid.km+(1|WinterYear), data=MigDis, REML=FALSE)

summary(waaC)

waaC2<-lmer(WingArea_mm~Sex+(1|WinterYear), data=MigDis, REML=FALSE)

summary(waaC2)

anova(waaC, waaC2, test="Chi")

confint(waaC)

# Analysis using linear models

wagmlC<-lm(WingArea_mm~Sex+dist.centroid.km+SOI, data=MigDis)

summary(wagmlC)

confint(wagmlC)

## WingLength

# Mixed-effects models

wlaC<-lmer(WingLength_mm~Sex+dist.centroid.km+(1|WinterYear), data=MigDis, REML=FALSE)

summary(wlaC)

wlaC2<-lmer(WingLength_mm~Sex+(1|WinterYear), data=MigDis, REML=FALSE)

summary(wlaC2)

anova(wlaC, wlaC2, test="Chi")

confint(wlaC)

# Analysis using linear models

wlgmlC<-lm(WingLength_mm~Sex+dist.centroid.km+SOI, data=MigDis)

summary(wlgmlC)

confint(wlgmlC)

## WingRoundness

# Mixed-effects models

r2aC<-lmer(Roundness~Sex+dist.centroid.km+(1|WinterYear), data=MigDis, REML=FALSE)

summary(r2aC)

r2aC2<-lmer(Roundness~Sex+(1|WinterYear), data=MigDis, REML=FALSE)

summary(r2aC2)

anova(r2aC, r2aC2, test="Chi")

confint(r2aC)

# Analysis using linear models

r2gmlC<-lm(Roundness~Sex+dist.centroid.km+SOI, data=MigDis)

summary(r2gmlC)

confint(r2gmlC)

## AspectRatio

# Mixed-effects models

araC<-lmer(AspectRatio~Sex+dist.centroid.km+(1|WinterYear), data=MigDis, REML=FALSE)

summary(araC)

araC2<-lmer(AspectRatio~Sex+(1|WinterYear), data=MigDis, REML=FALSE)

summary(araC2)

anova(araC, araC2, test="Chi")

confint(araC)

# Analysis using linear models

argmlC<-lm(AspectRatio~Sex+dist.centroid.km+SOI, data=MigDis)

summary(argmlC)

confint(argmlC)

################################################################################

################# Analyses with temperature

### Data Analysis using HIGH-POINT MIGRATION DISTANCE

## WingArea

# Mixed-effects models

waaHt<-lmer(WingArea_mm~Sex+temp.high.point+dist.high.point.km+(1|WinterYear), data=MigDis, REML=FALSE)

summary(waaHt)

waaH2t<-lmer(WingArea_mm~Sex+temp.high.point+(1|WinterYear), data=MigDis, REML=FALSE)

summary(waaH2t)

anova(waaHt, waaH2t, test="Chi")

confint(waaHt)

vif.mer(waaHt)

# Analysis using linear models

wagmlHt<-lm(WingArea_mm~Sex+temp.high.point+dist.high.point.km+SOI, data=MigDis)

summary(wagmlHt)

confint(wagmlHt)

vif(wagmlHt)

## WingLength

# Mixed-effects models

wlaHt<-lmer(WingLength_mm~Sex+temp.high.point+dist.high.point.km+(1|WinterYear), data=MigDis, REML=FALSE)

summary(wlaHt)

wlaH2t<-lmer(WingLength_mm~Sex+temp.high.point+(1|WinterYear), data=MigDis, REML=FALSE)

summary(wlaH2t)

anova(wlaHt, wlaH2t, test="Chi")

confint(wlaHt)

vif.mer(wlaHt)

# Analysis using linear models

wlgmlHt<-lm(WingLength_mm~Sex+temp.high.point+dist.high.point.km+SOI, data=MigDis)

summary(wlgmlHt)

confint(wlgmlHt)

vif(wlgmlHt)

## WingRoundness

# Mixed-effects models

r2aHt<-lmer(Roundness~Sex+temp.high.point+dist.high.point.km+(1|WinterYear), data=MigDis, REML=FALSE)

summary(r2aHt)

r2aH2t<-lmer(Roundness~Sex+temp.high.point+(1|WinterYear), data=MigDis, REML=FALSE)

summary(r2aH2t)

anova(r2aHt, r2aH2t, test="Chi")

confint(r2aHt)

vif.mer(r2aHt)

# Analysis using linear models

r2gmlHt<-lm(Roundness~Sex+temp.high.point+dist.high.point.km+SOI, data=MigDis)

summary(r2gmlHt)

confint(r2gmlHt)

vif(r2gmlHt)

## AspectRatio

# Mixed-effects models

araHt<-lmer(AspectRatio~Sex+temp.high.point+dist.high.point.km+(1|WinterYear), data=MigDis, REML=FALSE)

summary(araHt)

araH2t<-lmer(AspectRatio~Sex+temp.high.point+(1|WinterYear), data=MigDis, REML=FALSE)

summary(araH2t)

anova(araHt, araH2t, test="Chi")

confint(araHt)

vif.mer(araHt)

# Analysis using linear models

argmlHt<-lm(AspectRatio~Sex+temp.high.point+dist.high.point.km+SOI, data=MigDis)

summary(argmlHt)

confint(argmlHt)

vif(argmlHt)

## Data Analysis using CENTROID MIGRATION DISTANCE

## WingArea

# Mixed-effects models

waaCt<-lmer(WingArea_mm~Sex+temp.centroid+dist.centroid.km+(1|WinterYear), data=MigDis, REML=FALSE)

summary(waaCt)

waaC2t<-lmer(WingArea_mm~Sex+temp.centroid+(1|WinterYear), data=MigDis, REML=FALSE)

summary(waaC2t)

anova(waaCt, waaC2t, test="Chi")

confint(waaCt)

vif.mer(waaCt)

# Analysis using linear models

wagmlCt<-lm(WingArea_mm~Sex+temp.centroid+dist.centroid.km+SOI, data=MigDis)

summary(wagmlCt)

confint(wagmlCt)

vif(wagmlCt)

## WingLength

# Mixed-effects models

wlaCt<-lmer(WingLength_mm~Sex+temp.centroid+dist.centroid.km+(1|WinterYear), data=MigDis, REML=FALSE)

summary(wlaCt)

wlaC2t<-lmer(WingLength_mm~Sex+temp.centroid+(1|WinterYear), data=MigDis, REML=FALSE)

summary(wlaC2t)

anova(wlaCt, wlaC2t, test="Chi")

confint(wlaCt)

vif.mer(wlaCt)

# Analysis using linear models

wlgmlCt<-lm(WingLength_mm~Sex+temp.centroid+dist.centroid.km+SOI, data=MigDis)

summary(wlgmlCt)

confint(wlgmlCt)

vif(wlgmlCt)

## WingRoundness

# Mixed-effects models

r2aCt<-lmer(Roundness~Sex+temp.centroid+dist.centroid.km+(1|WinterYear), data=MigDis, REML=FALSE)

summary(r2aCt)

r2aC2t<-lmer(Roundness~Sex+temp.centroid+(1|WinterYear), data=MigDis, REML=FALSE)

summary(r2aC2t)

anova(r2aCt, r2aC2t, test="Chi")

confint(r2aCt)

vif.mer(r2aCt)

# Analysis using linear models

r2gmlCt<-lm(Roundness~Sex+temp.centroid+dist.centroid.km+SOI, data=MigDis)

summary(r2gmlCt)

confint(r2gmlCt)

vif(r2gmlCt)

## AspectRatio

# Mixed-effects models

araCt<-lmer(AspectRatio~Sex+temp.centroid+dist.centroid.km+(1|WinterYear), data=MigDis, REML=FALSE)

summary(araCt)

araC2t<-lmer(AspectRatio~Sex+temp.centroid+(1|WinterYear), data=MigDis, REML=FALSE)

summary(araC2t)

anova(araCt, araC2t, test="Chi")

confint(araCt)

vif.mer(araCt)

# Analysis using linear models

argmlCt<-lm(AspectRatio~Sex+temp.centroid+dist.centroid.km+SOI, data=MigDis)

summary(argmlCt)

confint(argmlCt)

vif(argmlCt)

################################################################################

# end
